# Clinical and endoscopic characteristics of patients undergoing gastrointestinal endoscopic procedures in Egypt: a nationwide multicenter study

**DOI:** 10.1186/s12876-024-03262-3

**Published:** 2024-05-28

**Authors:** Mohamed Elbadry, Fathiya El-Raey, Mohamed Alboraie, Mohamed Abdel-Samiee, Doaa Abdeltawab, Mohammed Hussien Ahmed, Ahmed F Sherief, Ahmed Eliwa, Mina Tharwat, Amira Abdelmawgod, Ossama Ashraf Ahmed, Eman Abdelsameea, Aya Mahros, Abdelmajeed M Moussa, Alshaimaa Eid, Khaled Raafat, Ahmed Yousef, Saad A. S. Rafea, Youssef Alazzaq, Mohamed Mare’y, Ahmed Abdelaziz, El Sayed Abouzid Ibrahim, Waleed A Abd El Dayem, Ahmed A Abdelmoati, Ahmed Tawheed, Mohammad Amer, Samy Zaky, Mohamed El-Kassas

**Affiliations:** 1 Endemic Medicine Department, Faculty of Medicine, Helwan University, Ain Helwan, Cairo, 11795 Egypt; 2 Hepatogastroenterology and Infectious Diseases Department, Al-Azhar University, Damietta, Egypt; 3 Internal Medicine Department, Al-Azhar University, Cairo, Egypt; 4 Hepatology and Gastroenterology Department, National Liver Institute, Menoufia University, Shebin El-Kom, Egypt; 5 Tropical Medicine and Gastroenterology Department, Assiut University, Assiut, Egypt; 6 Hepatology, Gastroenterology, and Infectious Diseases Department, Kafrelsheikh University, Kafrelsheikh, Egypt; 7 Tropical Medicine Department, Ain Shams University, Cairo, Egypt; 8 Tropical Medicine and Gastroenterology Department, Aswan University, Aswan, Egypt; 9 Internal Medicine Department, Ain Shams University, Cairo, Egypt; 10 Hepatogastroenterolgy and Infectious Diseases Department, AL-Azhar University, Cairo, Egypt; 11 Public Health and Community Medicine Department, Damietta Faculty of Medicine, Al-Azhar University, Damietta, Egypt; 12 Damietta Faculty of Medicine, Al-Azhar University, Damietta, Egypt; 13 Internal Medicine Department, Al-Azhar University, Damietta, Egypt; 14 Tropical Medicine Department, Zagazig University, Zagazig, Egypt; 15 Hepatology & Gastroenterology, Shebin Teaching Hospital, Shebin El Kom, Egypt

**Keywords:** Esophagogastroduodenoscopy (EGD), Colonoscopy, Endoscopy, Egypt, Indications

## Abstract

**Background:**

Egypt faces a significant public health burden due to chronic liver diseases (CLD) and peptic ulcer disease. CLD, primarily caused by Hepatitis C virus (HCV) infection, affects over 2.9% of the population nationwide, with regional variations. Steatotic liver disease is rapidly emerging as a significant contributor to CLD, especially in urban areas. Acid-related disorders are another widespread condition that can significantly impact the quality of life. These factors and others significantly influence the indications and findings of gastrointestinal endoscopic procedures performed in Egypt.

**Aim:**

We aimed to evaluate the clinico-demographic data, indications, and endoscopic findings in Egyptian patients undergoing gastrointestinal endoscopic procedures in various regions of Egypt.

**Methods:**

This study employed a retrospective multicenter cross-sectional design. Data was collected from patients referred for gastrointestinal endoscopy across 15 tertiary gastrointestinal endoscopy units in various governorates throughout Egypt.

**Results:**

5910 patients aged 38–63 were enrolled in the study; 75% underwent esophagogastroduodenoscopy (EGD), while 25% underwent a colonoscopy. In all studied patients, the most frequent indications for EGD were dyspepsia (19.5%), followed by hematemesis (19.06%), and melena (17.07%). The final EGD diagnoses for the recruited patients were portal hypertension-related sequelae (60.3%), followed by acid-related diseases (55%), while 10.44% of patients had a normally apparent endoscopy. Male gender, old age, and the presence of chronic liver diseases were more common in patients from upper than lower Egypt governorates. Hematochezia (38.11%) was the most reported indication for colonoscopy, followed by anemia of unknown origin (25.11%). IBD and hemorrhoids (22.34% and 21.86%, respectively) were the most prevalent diagnoses among studied patients, while normal colonoscopy findings were encountered in 18.21% of them.

**Conclusion:**

This is the largest study describing the situation of endoscopic procedures in Egypt. our study highlights the significant impact of regional variations in disease burden on the utilization and outcomes of GI endoscopy in Egypt. The high prevalence of chronic liver disease is reflected in the EGD findings, while the colonoscopy results suggest a potential need for increased awareness of colorectal diseases.

## Introduction

Over the last decades, esophagogastroduodenoscopy (EGD) and colonoscopy have become essential tools in diagnosing, treating, and screening gastrointestinal diseases. Upper and lower endoscopies are widely used and relatively costly [1]. The symptoms of upper and lower gastrointestinal diseases are various and very common [2, 3]. esophagogastroduodenoscopy and ileocolonoscopic examination yield an excellent modality for evaluating the underlying pathologies by enabling the direct visual observation of the mucosa of the upper and lower gastrointestinal tract (GIT) and facilitating urgent interventions. However, upper and lower GI endoscopies are invasive procedures requiring anesthesia [4]. Being invasive modalities, endoscopies may carry a risk of complications in addition to practice variation in the type of anesthesia from one center to another. Spontaneous ventilation without the patient’s intubated airway is safe and effective. Most anesthesia complications are respiratory and usually occur during an upper endoscopy, including apnoea, laryngospasm, and airway obstruction. Most problems are resolved after the withdrawal of the endoscope and positive pressure ventilation with a tightly fitting mask [5, 6]. Other complications related to endoscopy are likely to rise due to the overuse of endoscopy for therapeutic procedures and the increased complexity of endoscopic techniques. Informed patient consent should be obtained before the procedure. Avoidance of endoscopic adverse events is based on knowledge of the risk of complications and their mechanisms of occurrence [7]. We aimed to collect and analyze the recently available data from several endoscopic centers of different governorates to draw a realistic map of diagnostic and therapeutic gastrointestinal endoscopies in Egypt and introduce evidence-based recommendations to help the local health authority optimize endoscopic management strategies.

## Methods

This study adopted a retrospective multicenter cross-sectional design. Data was collected from patients referred for endoscopic evaluation at tertiary gastrointestinal units within university hospitals across various Egyptian governorates from October 2016 to September 2021. Patients who underwent EGD and/or colonoscopy with their complete medical records available were included in the study, while those with incomplete medical records were excluded.

Egypt is divided into 27 governorates, with the four urban governorates (Cairo, Alexandria, Port Said, and Suez) having no rural population. Nine of these governorates are located in the Nile Delta (Lower Egypt), nine are located in the Nile Valley (Upper Egypt), and the remaining five Frontier Governorates are located on the eastern and western boundaries of Egypt [8]. Each of the other 23 governorates is subdivided into urban and rural areas.

### Patient’s variables

Clinical and epidemiological characteristics were collected from medical records in the form of age, sex, body mass index (BMI), associated co-morbidities such as diabetes mellitus (DM), hypertension, chronic obstructive pulmonary disease (COPD), chronic kidney disease (CKD), chronic liver disease (CLD), ischemic heart disease (IHD). Also, we recorded history of smoking, alcohol intake, specific drug history (e.g., non-steroidal anti-inflammatory drugs (NSAIDs), anticoagulants or proton pump inhibitors (PPI)) and history of GIT diseases like inflammatory bowel disease (IBD) or colorectal carcinoma (CRC). Additionally, indications of endoscopy were recorded. Several laboratory tests were collected from the patient’s medical records during the study: Complete blood count (CBC), Liver function tests, Serum alanine transaminase (ALT), and aspartate transaminase (AST). Normal ranges were set at 41 IU/L for ALT and 37 IU/L for AST. Total serum bilirubin: The normal range was 0.5-1 mg/dl. Serum albumin: The normal range was 3.5–5.5 mg/L. Coagulation tests: Prothrombin time (PT) and concentration (PC) assess blood clotting function. Normal values were set at 12–14 s for PT, up to 75% for PC, and an INR (international normalized ratio) up to 1. Fecal occult blood test [FOBT]): This test detects hidden blood in the stool, which can signify gastrointestinal bleeding. A normal FOBT result was defined as less than 2 to 3 mg/gm. Fecal Calprotectin: This test measures calprotectin levels in the stool, a protein elevated during intestinal inflammation. A normal fecal calprotectin level was to be less than 50 µg/mg. Data of upper and lower GI endoscopic reports obtained, including the mucosal and vascular pattern, presence or absence of congestion, hyperemia, erythema, erosions, ulceration, spontaneous bleeding and/or nodule, polyps, or masses. Biopsies and/or duodenal aspirates were taken when indicated.

### Statistical analysis

Analysis used SPSS version 25.0 (IBM SPSS Statistics for Windows, Armonk, NY: IBMCorp., USA). Mean ± SD was used for quantitative variables, and frequency and percentage were used for qualitative variables. Mann-Whitney and Wilcoxon’s tests were used to assess the differences in means of quantitative nonparametric variables. Chi-square and Fisher’s Exact tests were used to assess differences in the frequency of qualitative variables. The statistical methods assumed a significance level of *p* < 0.05 and a highly significant level of *p* < 0.00.

## Results

This study enrolled a total of 5,910 patients between the ages of 38 and 63 years. Males comprised the larger portion of the study population (66.57%) compared to females (33.43%). Geographically, 1,298 patients (21.98%) originated from Upper Egypt governorates, while the majority (78.03%) resided in Lower Egypt governorates. Regarding EGD, a total of 4,433 patients underwent EGD. Of these, 3,602 (81.2%) resided in Lower Egypt, and 831 (17.8%) resided in Upper Egypt. Table (1) presents a breakdown of patient characteristics according to residence (Upper vs. Lower Egypt), including smoking history, alcohol consumption, and medication use (anticoagulants, NSAIDs, proton pump inhibitors). Analysis of the timing of EGD revealed that early endoscopy within 24 h was prevalent among patients from Upper Egypt. On the other hand, elective endoscopy (> 24 h) was prevalent among patients from Lower Egypt (P value < 0.001). Regarding indication for EGD:

**Table 1:** Socio-demographic data and clinical indications of endoscopy in patients underwent EGD

Variables			Upper Egypt 831(18.7%)	Lower Egypt 3602(81.2%)	Total 4433(100%)	*P*. Value
**Age (Mean ± SD)**			50.21 **±** 16.49	48.58 **±** 13.31	48.87 **±** 13.95	**0.001** $\bar T$
**Age (Median [IQR])**			53(38–63)	50(40–58)	50(40–59)	
**Sex (N [%])**		**Male**	424(51.1%)	2146(59.5)	2570 (57.9%)	0.462*
		**Female**	407(48.9%)	1456(40.5)	1863 (42.1%)	
**Smoking**		**Yes**	327(39.35%)	633(17.57%)	960(21.7%)	0.001*
**Alcohol**		**Yes**	12(1.4%)	10(0.27%)	22(0.49%)	0.001*
**Drug History**	**NSAIDs**	**Yes**	207(24.9%)	470(13.04%)	677(15.3%)	0.001*
	**Anticoagulants**	**Yes**	16(1.9%)	14(0.38%)	30 (0.7%)	0.001*
	**PPI**	**Yes**	309(37.2%)	961(26.7%)	1270(28.6%)	0.001*
**Indications**	**Dyspepsia**	**Yes**	56(6.7%)	809(22.4%)	865(19.5%)	0.001*
	**Anemia**	**Yes**	17(2.04%)	337(9.3%)	354(7.9%)	0.001*
	**Dysphagia**	**Yes**	11(1.3%)	105(2.9%)	116(2.6%)	0.01*
	**Hematemesis**	**Yes**	326(39.2%)	519(14.4%)	845(19.06%)	0.001*
	**Melena**	**Yes**	431(51.8%)	326(9.05%)	757(17.07%)	0.001*
	**Weight Loss**	**Yes**	7(0.8%)	102(2.8%)	109(2.4%)	0.003*
	**Odynophagia**	**Yes**	0(0.0)	6(0.1%)	6(0.13%)	0.239*
	**Heartburn**	**Yes**	47(5.6%)	471(13.1%)	518(11.7%)	0.001*
	**Nausea**	**Yes**	36(4.3%)	158(4.4%)	194(4.4%)	0.945*
	**Vomiting**	**Yes**	51(6.1%)	328(9.1%)	379(8.5%)	0.006*
	**Diarrhea**	**Yes**	6(0.7%)	26(0.7%)	32(0.72%)	1.000*
	**Abdominal Pain**	**Yes**	70(8.4%)	651(18.1%)	721(16.3%)	0.001*
	**Follow up of varices**	**Yes**	152(18.2%)	708(19.6%)	860(19.4%)	0.370*
**Past medical history**	**DM**	**Yes**	162(19.5%)	555(15.4%)	717(16.2%)	0.637*
	**HTN**	**Yes**	219(26.3%)	325(9.02%)	544(12.3%)	0.001*
	**CKD**	**Yes**	35(4.2%)	21(0.58%)	56(1.2%)	0.001*
	**CLD**	**Yes**	330(39.7%)	869(24.1%)	1199(27.05%)	0.001*
	**IHD**	**Yes**	39(4.7%)	31(0.86%)	70(1.6%)	0.001*
	**COPD**	**Yes**	17 (2.04%)	19(0.52%)	36(0.8%)	0.001*
	**Surgical operation**	**Yes**	180(21.7%)	381(10.6%)	561(12.6%)	0.001*
**Timing of EGD**	**Elective (> 24 h)**		304(36.5%)	2305(63.9%)	2609(58.8%)	0.034*
	**Early (< 24 h)**		527(63.5%)	1297(36.1%)	1824(41.2%)	0.023*

P value is significant < 0.05, (*) Pearson Chi-Square Test

Missing (age) = 36 (33 Upper – 3 Lower)

Missing (sex) = 976 (22.01%)

Missing (DM, HTN, CKD, IHD, COPD) = 645 Cases

Diagnostic EGD was more commonly performed than therapeutic EGD in both patient groups (detailed in Table 2). Dyspepsia emerged as the most common overall indication for EGD (19.5%), followed by hematemesis (19.06%) and then melena (17.07%). Table 1 also presents a more specific analysis based on residence in Upper Egypt: Melena was the most frequent indication (51.3%). However, in lower Egypt, dyspepsia was the most common indication of EGD (22.4%). The use of sedation medications prior to EGD differed significantly between the two regions (p-value < 0.001). In Lower Egypt, A significantly lower proportion of patients (88.2%) received sedation before the procedure compared to Upper Egypt (41%). Also, lower Egypt had a lower percentage of patients receiving dual sedation medications (propofol and midazolam) compared to Upper Egypt. The majority of patients in both regions received either midazolam or propofol alone. Regarding EGD Findings: Portal hypertension-related complications (60.3%) were the most prevalent final diagnosis, followed by acid-related disorders (55%) among all patients. Table 2 provides a further breakdown of EGD diagnoses by residence: Upper Egypt, Portal hypertension (76.8%), and acid-related disorders (68.2%) were the most common diagnoses. Lower Egypt: Portal hypertension (56.6%) and acid-related disorders (52.2%) were still frequent diagnoses but at a lower prevalence compared to Upper Egypt. Normally apparent endoscopy (no significant findings) was observed in 10.44% of patients. There were statistically significant differences (p-value < 0.001) in all diagnostic categories between Upper and Lower Egypt. H. Pylori Infection: Biopsies were obtained from 653 patients (14.73%) for histological examination. H. pylori infection was identified in 318 (48.7%) of biopsied patients. H. pylori infection was more prevalent in Lower Egypt compared to Upper Egypt (p-value < 0.001), as detailed in Table 2. Upper Egypt: Biopsies were taken from 8.4% of patients (70/831), with a positive H. pylori detection rate of 45.7% (32/70). Lower Egypt: Biopsies were obtained from 16.2% of patients (583/3602), and 49.05% (286/583) tested positive for H. pylori.

**Table 2:** Recorded EGD diagnosis

Variables		Upper Egypt 831(18.7%)	Lower Egypt 3602(81.2%)	Total 4433(100%)	*P*. Value
Esophagus, Stomach, Duodenum	Normal	14(1.7%)	449(12.46%)	463(10.44%)	0.001*
	**Portal Hypertension**	638 (76.8%)	2037 (56.6%)	2675 (60.3%)	
	**Motility Disorders**	5 (0.6%)	18 (0.5%)	23 (0.5%)	
	**Acid Related Disorders**	567 (68.2%)	1882 (52.2%)	2449 (55.2%)	
	**Malignancy**	15 (1.8%)	177 (5%)	192 (4.3%)	
	**Others**	68 (8.1%)	429 (12%)	497 (11.2%)	
**Biopsy**	**Yes**	70(8.4%)	583(16.2%)	653(14.73%)	0.001*
	**H.Pylori positive**	32 (45.7%)	286 (49.05%)	318 (48.7%)	0.001*

P value is significant < 0.05, (*) Pearson Chi-Square Test

Normal

Portal hypertension: esophageal varices, gastric varices, portal hypertensive gastropathy and portal hypertensive Duodenopathy

Motility disorders include: (Achalasia)

Acid related disorders: (GERD, gastric ulcer, duodenal ulcer, H. pylori and all types of gastropathy and Duodenopathy including erosive, NSAIDs or hemorrhagic) Malignancy: Barrett’s and malignancy at any part of upper GIT

### Results of colonoscopy

Almost 1010 (68.38%) of the 1477 patients were from Lower Egypt, while 467 (31.62%) were from Upper Egypt. Older patients (44.85 ± 24.56) were from Upper Egypt than Lower Egypt (47.64 ± 15.25). Patients from Lower Egypt were found to use significantly more NSAIDs and PPI than those from Upper Egypt. Most patients from Upper Egypt were hypertensive and had chronic liver diseases. Hematochezia was the most common (38.11%) indication for colonoscopy among all included patients, followed by anemia of unexplained cause (25.11%) (Table 3). Laboratory characteristics of patients subjected to colonoscopy were analyzed and revealed significant differences in HB level between patients from Upper and Lower Egypt. Fecal occult blood test was not done in 73% of the patients, while it was positive in 13.5%. The fecal calprotectin test was not done in 72.8% of patients, while it was abnormal in 12% (Table 4).

**Table 3:** Socio-demographic data and clinical indications in patients underwent colonoscopy

Variables				Upper Egypt 467(31.5%)	Lower Egypt 1010(68.2%)	Total 1477(100%)	*P*. Value
**Age (Mean ± SD)**				47.64 **±** 15.25	44.85 **±** 24.56	45.75 **±** 22.05	**0.001$**
**Age (Median [IQR])**				48(35–60)	45(33–55)	45(34–56)	
**Sex**			**Male**	288(61.6%)	631(62.47%)	919(62.22%)	0.767*
			**Female**	179(38.32%)	379(37.52%)	558(37.77%)	
**Smoking**			**Yes**	134(28.69%)	275(27.22%)	409(27.69%)	0.558*
**Alcohol**			**Yes**	4(0.85%)	5(0.49%)	9(0.60%)	0.476$$\bar T$$
**Family History**	**CRC**		**Yes**	15(3.21%)	58(5.74%)	73(4.94%)	0.037*
	**IBD**		**Yes**	7(1.49%)	27(2.67%)	34(2.30%)	0.162*
**Drug History**	**NSAIDs**		**Yes**	17(3.64%)	87(8.61%)	104(7.04%)	0.001*
	**Steroids**		**Yes**	9(1.92%)	27(2.67%)	36(2.43%)	0.387*
	**Antibiotics**		**Yes**	2(0.42%)	4(0.39%)	6(0.40%)	1.000$$\bar T$$
	**Anticoagulants**		**Yes**	0 (0.0)	3(0.29%)	3(0.20%)	0.556$$\bar T$$
	**PPI**		**Yes**	5(1.07%)	58(5.74%)	63(4.26%)	0.001*
**Medical History**		**DM**	**Yes**	66(14.31%)	169(16.37%)	235(15.91%)	0.204*
		**HTN**	**Yes**	90(19.27%)	137(13.56%)	227(15.36%)	0.005*
		**CKD**	**Yes**	7(1.49%)	9(0.89%)	16(1.08%)	0.294*
		**CLD**	**Yes**	5(1.07%)	34(3.36%)	39(2.64%)	0.010*
		**IHD**	**Yes**	13(2.78%)	16(1.58%)	29(1.96%)	0.122*
		**Hematochezia**	**Yes**	270(57.81%)	294(29.10%)	564(38.11%)	0.001*
		**Unexplained anemia**	**Yes**	105(22.48%)	266(26.33%)	371(25.11%)	0.112*
		**Abdominal Pain**	**Yes**	75(16.05%)	280(27.72%)	355(24.03%)	0.001*
		**Constipation**	**Yes**	32(6.85%)	178(18.51%)	210(14.21%)	0.001*
		**Weight Loss**	**Yes**	25(5.53%)	104(10.29%)	129(8.73%)	0.020*
		**Diarrhea**	**Yes**	24(5.31%)	128(1.78%)	152(10.29%)	0.001*
		**Abdominal Mass**	**Yes**	15(3.21%)	6(0.59%)	21(1.42%)	0.001*
		**Altered Bowel Habits**	**Yes**	7(1.49%)	33(3.26%)	40(2.70%)	0.058*
		**Defecation Disorders**	**Yes**	3(0.64%)	113(11.18%)	116(7.85%)	**0.001** $$\bar T$$
		**Melena**	**Yes**	0 (0.0)	7(0.69%)	7(0.47%)	**0.105** $$\bar T$$
		**Follow up**	**Yes**	28 (5.99%)	221(21.88%)	249(16.85%)	**0.334** $$\bar T$$

P value is significant < 0.05, (*) Pearson Chi-Square Test, ($\bar T$) Fischer’s Exact test

**Table 4:** Laboratory data of patients subjected to colonoscopy

Variables	Upper Egypt 467(31.5%)	Lower Egypt 1010(68.2%)	Total 1477(100%)	*P*. Value
**HGB (**g/Dl)	10.9 **±** 1.5	11.3 **±** 1.7	11.1 **±** 1.6	**0.041#**
**WBC** (×10^3^) /cmm	6.3 **±** 1.9	6.7 **±** 1.3	6.4 **±** 1.7	**0.226**
**Platelet**(×103) /cmm	208.3 **±** 49.5	209.7 **±** 41.5	209.1 **±** 45.4	**0.850**
**ALT**(IU/L)	49.7 **±** 27.1	50.3 **±** 17.8	50.1 **±** 22.7	**0.873**
**AST**(IU/L)	48.4 **±** 30.7	49.0 **±** 18.4	48.7 **±** 25.1	**0.877**
**Albumin**(g/L)	4.3 **±** 0.4	4.3 **±** 0.3	4.36 **±** 0.4	**0.705**
**PC (%)**	89.7 **±** 8.3	87.8 **±** 7.2	88.7 **±** 7.8	**0.146**
**INR**	1.08 **±** 0.07	1.11 **±** 0.12	1.1 **±** 0.08	**0.062**
**FOBT**( mg/gm)				
Positive	74(15.8%)	126(12.4%)	200(13.5%)	0.001*
Negative	21(4.3%)	180(17.8%)	201(13.5%)	
Not Done	372(79.9%)	704(69.8%)	1076(73.0%)	
**Fecal Calprotectin** µg/mg				
Normal	39 (8.4%)	186 (18.4%)	225 (15.2%)	0.001*
Abnormal	37 (7.9%)	140 (13.9%)	177 (12.0%)	
Not Done	391(83.7%)	684 (67.7%)	1075(72.8%)	

P value is significant < 0.05, (*) Pearson Chi-Square Test, (#) Independent t test

**Fig. 1:**
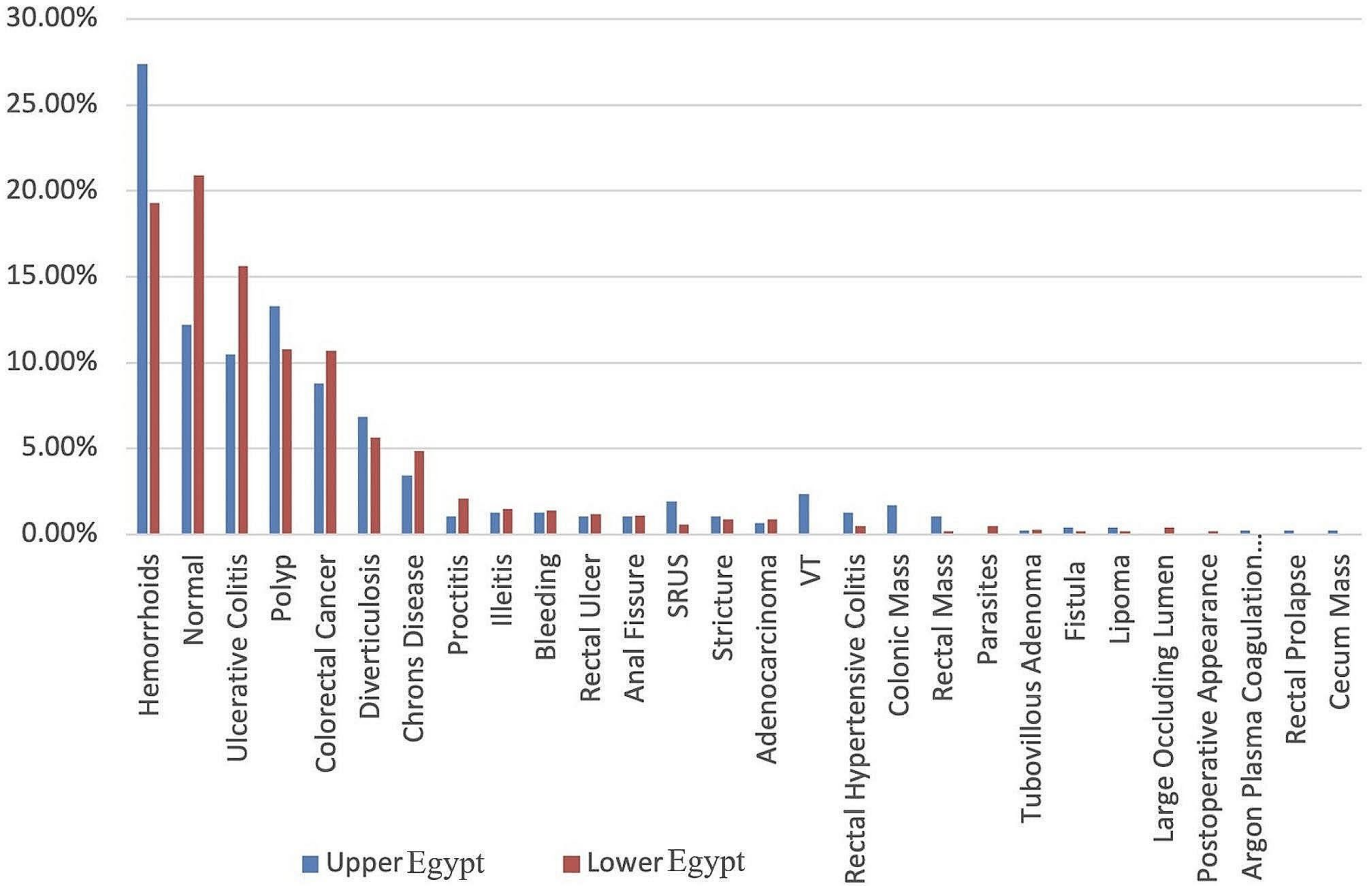
Common final colonoscopy diagnosis

Out of 1477 conducted colonoscopies, 50.5% of patients were sedated with propofol, 72.88% of patients were prepared with Poly Ethylene Glycol (PEG), the mean total procedure time was (23.22 ± 8.18) minutes with a significantly longer duration of colonoscopy among patients from Upper than Lower Egypt. The mean withdrawal time of colonoscopy was (9.65 ± 5.12) minutes, with a significantly longer duration of withdrawal among patients from Upper than Lower Egypt. Less than 1% (18/1477) of enrolled patients recorded complications related to colonoscopy in the form of bleeding, followed by perforation and drug allergy. There were no discernible differences between patients regarding complications associated with colonoscopies (Table 5). When all patients’ final colonoscopy diagnoses were analyzed, it became clear that IBD (22.34%), hemorrhoids (21.86%), and normal apparent colonoscopy (18.21%) were the most common diagnoses (Fig. 1).

**Table 5:** Sedation, colon preparation, and some colonoscopic features for colonoscopy

Variables		Upper Egypt 467(31.5%)	Lower Egypt 1010(68.2%)	Total 1477(100%)	*P*. Value
**Sedation**	**No**	180(38.54%)	26(2.57%)	206(13.94%)	0.001*
	**Propofol**	189(40.47%)	557(55.14%)	746(50.5%)	
	**Midazolam**	98(20.98%)	145(14.35%)	243(16.45%)	
	**Pethidine**	0(0.0)	150(14.85%)	150(10.15%)	
	**Propofol + Midazolam**	0(0.0)	132(13.06%)	132(8.93%)	
**Colon Preparation**	**PEG**	188(40.25%)	857(84.79%)	1045(72.88%)	0.001*
	**Non-PEG**	279(59.74%)	153(15.02%)	432(27.11%)	
**Difficult procedure**	**Yes**	69 (14.77%)	219 (21.68%)	288 (19.49%)	0.002*
	**No**	398 (85.22%)	791(78.31%)	1189(80.50%)	
**Need for abdominal compression**	**Yes**	159 (34.04%)	481 (47.62%)	640 (43.33%)	0.001*
	**No**	308 (65.95%)	529 (52.37%)	837 (56.66%)	
**Total procedure time “Minutes”**		25.98 ± 8.03	22.35 ± 8.04	23.22 ± 8.18	**0.001#**
**Withdrawal time “Minutes”**		12.08 ± 6.18	8.90 ± 4.50	9.65 ± 5.12	**0.001#**
**Type of used Endoscopy**	**Pentax**	165(98.8%)	343(79.2%)	508(84.6%)	**0.001** $\bar T$
	**Olympus**	2(1.2%)	90(20.8%)	92(15.3%)	
	**Fuji**	0(0.0)	0(0.0)	0(0.0)	
**Complications**	**Perforation**	1(0.21%)	2(0.19%)	3(0.20%)	1.000$\bar T$
	**Bleeding**	3(0.64%)	9(0.89%)	12(0.81%)	0.762$\bar T$
	**Allergy**	1(0.21%)	2(0.19%)	3(0.20%)	1.000$\bar T$

P value is significant < 0.05, (*) Pearson Chi-Square Test, (#) Independent t test, ($\bar T$) Fischer’s Exact test, Missing Upper = 300 Patients, Lower = 577 Patients, Total = 877 Patients

## Discussion

Digestive tract diseases significantly affect millions of people worldwide and have an economic healthcare burden [7]. Over the last few years, clinical considerations for managing gastrointestinal tract diseases have changed dramatically. These changes due to endoscopy have evolved over time, fulfilling a widening diagnostic and therapeutic remit [9]. 

In this study, elective UGE (> 24 h) was more among patients from Lower Egypt. By place of residence in Egypt governorates, the proportion with a sample tested for HCV was highest in rural Lower Egypt (93%) followed closely by rural Upper Egypt (92%) and lowest in the Urban Governorates (77%) and the Frontier Governorates [8]. (The rate of progression to cirrhosis is estimated at 7% after 20 years of being infected with HCV [10]. Screening endoscopy is recommended in patients with cirrhosis to determine if they have varices at high risk of bleeding, which will require treatment with either non-selective beta-blockers (NSBB) or endoscopic variceal ligation (EVL) in order to prevent variceal hemorrhage, in accordance with the most recent American Association for the Study of Liver Diseases guidance [11]. 

Dyspepsia is represented mainly by symptoms of epigastric pain, burning, early satiety, bloating upper abdomen, fullness, or nausea. It reflects most of the population that seeks health care, and it has a substantial economic cost to patients and the health care system [12]. Dyspepsia symptoms often occur in positive Helicobacter pylori (H. pylori) individuals. The proportion of patients with dyspeptic symptoms was significantly higher in H. pylori-positive patients than in H. pylori-negative patients [13, 14]. Identification of common indication of endoscopy in Al-Kharj Province, KSA reported that dyspepsia was the common indication for EGD in 19.6% of patients [15]. In this study, dyspepsia was the commonest indication for EGD in about one-fifth (19.5%) of all patients, with more prevalence among patients from Lower Egypt. This geographical variation may be related to the relatively high prevalence of H. pylori infection in patients from Delta Egypt [16]. 

Early upper GI endoscopy within 24 h from presentation for patients with acute upper gastrointestinal bleeding (UGIB) is recommended [17]. Assessment of the endoscopic findings in patients presenting with acute UGIB in Upper Egypt revealed that portal hypertension was the most common and important cause of UGIB (55.5%), especially esophageal varices, which constitutes about 48.2% of the cases, either alone or with gastric varices [18]. This agreed with our finding, where early diagnostic endoscopy was more prevalent among patients from Upper Egypt. Also, this was attributed to another finding in the current study where portal hypertension-related sequelae like varices were the most common (60.3%) final diagnosis of EGD. The high prevalence of esophageal varices among Egyptian patients might be due to the high prevalence of viral hepatitis (HBV & HCV) related cirrhosis [19]. Variceal hemorrhage is a life-threatening complication of cirrhosis and is one of the clinical complications that define cirrhosis decompensation. Screening and surveillance of varices aims to identify patients with gastroesophageal varices at a high risk of bleeding so that prevention strategies can be implemented [20]. Egypt established an excellent model of care for HCV management. According to the WHO HCV elimination targets, Egypt appears to be “on track” for HCV elimination [21]. As the incidence of esophageal varices in Egyptian HCV patients is still high [22], follow-ups for cirrhotic patients treated with DAAs with special variceal screening and surveillance programs should be adopted in Egypt.

Colonoscopy has emerged as the preferred procedure for evaluating patients with lower gastrointestinal symptoms. Until now, there is no national screening program for colorectal cancer in Egypt, and most patients undergo colonoscopies for specific complaints. Bleeding per rectum was the most common indication for colonoscopy [23, 24]. In Egypt, from affiliated hospitals in the middle of the Nile Delta, evaluation of the patient characteristics and final diagnosis in patients subjected to colonoscopy reported that bleeding per rectum is the commonest indication for colonoscopy [25]. Similarly, in this study, hematochezia was the most common indication for colonoscopy (38.11%) among included patients. Assessment of colonoscopy in high-risk groups presented to the GIT Surgery Unit at Alexandria University Hospital as a screening and diagnostic method for early detection of CRC showed that 52% (104/200) complained of bleeding per rectum [26]. Analysis of the most common final colonoscopy diagnosis in this study showed that hemorrhoids, followed by ulcerative colitis and colorectal carcinoma, were the most prevalent diagnoses. A cross-sectional evaluation of the prevalence of internal hemorrhoids in patients undergoing colonoscopies for different indications conducted on 300 Egyptian patients concluded a wide prevalence of internal hemorrhoids (38.3%) in patients who underwent colonoscopy for various reasons [27]. Evaluation of the final diagnosis in patients subjected to colonoscopy by *Elbatea et al.* reported diagnosis of ulcerative colitis in 22%of cases in the middle of the Nile Delta [25]. IBD was a more prevalent diagnosis in Lower Egypt than in Upper Egypt (24.55% and 17.6%, respectively). However, such differences may be due to many factors that should be studied in community-based research. Key issues may be socioeconomic status, environmental conditions, and access to diagnostic and treatment facilities. In Europe, higher numbers of normal colonoscopies were observed in more than half of colonoscopies [28, 29]. In this study, about one-fifth of conducted colonoscopies were normal, with no evidence of organic disease. This finding was in accordance with *Elbatea et al.*. exclusion of organic disease of the colon in 28% of cases, and their final diagnosis was irritable bowel syndrome (IBS), thyrotoxicosis, or diabetic neuropathy in the middle of Nile Delta [25]. A study of the quality of colonoscopy procedures in Egypt found that colonoscopies were performed at high standards, as evidenced by high cecal intubation and low complication rates (0.1%).[30] In this study, the real-life colonoscopy practice was safe as the complications were reported in less than 1% of patients. Also, conscious sedation was used in most conducted colonoscopies with average total procedure and withdrawal time. These observations were in line with British Society of Gastroenterology guidelines, which account for a minimum withdrawal time of 6 min for negative procedures [31]. 

While this study offers valuable insights into GI procedures in Egypt, some limitations restrict its generalizability:

- **Data Source**: Relying on retrospective medical records introduces the possibility of missing or inaccurate information. Prospective studies could provide a more reliable picture.
- **Selection Bias**: Patients referred for endoscopy might not represent the entire population with GI issues, limiting generalizability to the whole country.
- **Study Setting**: Focusing solely on tertiary GI units excludes data from primary care and secondary hospitals, potentially missing variations in practice and patient presentations.
- **Limited Outcome Data**: The study primarily focused on indications, findings, and H. pylori status, with minimal data on treatment outcomes and long-term follow-up.
- **Geographic Scope**: While the study divided data by Upper and Lower Egypt, further regional breakdowns could reveal more specific variations.

Future studies addressing these limitations could provide a more comprehensive understanding of GI diseases and endoscopic practices across all healthcare levels in Egypt.

## Conclusions

This nationwide multicenter study, the largest of its kind in Egypt, investigated the clinical and endoscopic characteristics of patients undergoing gastrointestinal endoscopy. Our findings reveal distinct patterns between upper and lower Egypt, drawing a valuable map of GIT disease distribution across the country.

Key findings reveal:

- **EGD**: There is a high prevalence of chronic liver disease and peptic ulcer disease nationwide, with portal hypertensive gastropathy and acid-related diseases being the most frequent EGD diagnoses.
- **Regional Variations**: Upper Egypt sees more early endoscopies and has a higher burden of chronic liver diseases, while Lower Egypt has a higher prevalence of H. pylori infection and utilizes elective endoscopies more frequently.
- **Colonoscopy**: Hematochezia is the top reason for colonoscopy, followed by anemia of unknown origin. Fecal occult blood testing and fecal calprotectin testing are underutilized. IBD, hemorrhoids, and normal colonoscopy findings are the most common diagnoses. Notably, colonoscopy procedures take longer in Upper Egypt.

These findings highlight the importance of considering regional variations in disease burden and optimizing resource allocation for GI endoscopy services in Egypt. Future studies with a broader scope could further strengthen these observations and provide a more comprehensive picture of GI health in the country.

## Data Availability

The data that support the findings of this study are available from the corresponding author upon reasonable request.

## References

[1] Heading RC (1999). Prevalence of upper gastrointestinal symptoms in the general population: a systematic review. Scand J Gastroenterol Suppl.

[2] Sobieraj DM, Coleman SM, Coleman CI (2011). US prevalence of upper gastrointestinal symptoms: a systematic literature review. Am J Manag Care.

[3] Early DS, Ben-Menachem T, Decker GA, Evans JA, Fanelli RD, Fisher DA, Fukami N, Hwang JH, Jain R, Jue TL, Khan KM, Malpas PM, Maple JT, Sharaf RS, Dominitz JA, Cash BD, ASGE Standards of Practice Committee (2012). Appropriate use of GI endoscopy. Gastrointest Endosc.

[4] Early DS, Lightdale JR, Vargo JJ, Acosta RD, Chandrasekhara V, Chathadi KV, Evans JA, Fisher DA, Fonkalsrud L, Hwang JH, Khashab MA, Muthusamy VR, Pasha SF, Saltzman JR, Shergill AK, Cash BD, DeWitt JM, ASGE Standards of Practice Committee (2018). Guidelines for sedation and anesthesia in GI endoscopy. Gastrointest Endosc.

[5] Goudra BG, Singh PM, Penugonda LC, Speck RM, Sinha AC (2014). Significantly reduced hypoxemic events in morbidly obese patients undergoing gastrointestinal endoscopy: predictors and practice effect. J Anaesthesiol Clin Pharmacol.

[6] Rex DK, Heuss LT, Walker JA, Qi RA (2005). Trained registered nurses/endoscopy teams can administer propofol safely for endoscopy. Gastroenterology.

[7] Peery AF, Crockett SD, Barritt AS, Dellon ES, Eluri S, Gangarosa LM, Jensen ET, Lund JL, Pasricha S, Runge T, Schmidt M, Shaheen NJ, Sandler RS (2015). Burden of gastrointestinal, liver, and pancreatic diseases in the United States. Gastroenterology.

[8] El-Zanaty F (2009). Egypt Demographic and Health Survey 2008.

[9] Peery AF, Crockett SD, Murphy CC, Jensen ET, Kim HP, Egberg MD, Lund JL, Moon AM, Pate V, Barnes EL, Schlusser CL, Baron TH, Shaheen NJ, Sandler RS (2022). Burden and cost of gastrointestinal, liver, and pancreatic diseases in the United States: Update 2021. Gastroenterology.

[10] Thein HH, Yi Q, Dore GJ, Krahn MD (2008). Estimation of stage-specific fibrosis progression rates in chronic hepatitis C virus infection: a meta‐analysis and meta‐regression. Hepatology.

[11] Garcia-Tsao G, Abraldes JG, Berzigotti A (2017). Portal hypertensive bleeding in cirrhosis: risk stratification, diagnosis, and management: 2016 practice guidance by the American Association for the study of liver diseases. Hepatology.

[12] Du LJ, Chen BR, Kim JJ, Kim S, Shen JH, Dai N (2016). Helicobacter pylori eradication therapy for functional dyspepsia: systematic review and meta-analysis. World J Gastroenterol.

[13] Shimatani T, Inoue M, Iwamoto K (2005). Prevalence of Helicobacter pylori infection, endoscopic gastric findings and dyspeptic symptoms among a young Japanese population born in the 1970s. J Gastroenterol Hepatol.

[14] Kawamura Y, Funaki Y, Yoshimine T, Tamura Y, Yamamoto S, Izawa S, Hayakawa T, Ebi M, Murotani K, Ogasawara N, Sasaki M, Kasugai K (2019). Characteristics and predictive factor of Helicobacter pylori-Associated Functional Dyspepsia in Japanese patients. Digestion.

[15] Aldujayn M, Almuaythir A, Almousa O, Alqahtani N, Alhaqbani S, Alshaalan F, Aug (2018). A cross-sectional Assessment of indications and findings of Upper and Lower Gastrointestinal Endoscopy in Population of Al Kharj Province, KSA. Saudi J Med.

[16] Abdelmonem M, Elshamsy M, Wasim H, Shedid M, Boraik (2020). Epidemiology of Helicobacter pylori in Delta Egypt. Am J Clin Pathol.

[17] Barkun AN, Almadi M, Kuipers EJ, Laine L, Sung J, Tse F, Leontiadis GI, Abraham NS, Calvet X, Chan FKL, Douketis J, Enns R, Gralnek IM, Jairath V, Jensen D, Lau J, Lip GYH, Loffroy R, Maluf-Filho F, Meltzer AC, Bardou M (2019). Management of Nonvariceal Upper gastrointestinal bleeding: Guideline recommendations from the International Consensus Group. Ann Intern Med.

[18] El Badry M, Eltaweel N, Moussa A (2020). Endoscopic findings in patients with Upper Gastrointestinal Bleeding in Upper Egypt: a single centre study. Afro-Egyptian J Infect Endemic Dis.

[19] Gouda Mkamel, Esmat G, Doss W. Acute upper gastrointestinal bleeding in Kasr ElAini gastrointestinal endoscopy unit in the last 10 years. cairo univ. 2002; https://www.cu.edu.eg/thesis_pdf/7125 Thesis_Acute upper gastrointestinal bleeding in.pdf.

[20] Jakab SS, Garcia-Tsao G. Screening and Surveillance of Varices in Patients With Cirrhosis. Clin Gastroenterol Hepatol. 2019;17(1):26–29. doi: 10.1016/j.cgh.2018.03.012. Epub 2018 Mar 15. Erratum in: Clin Gastroenterol Hepatol. 2019 Apr;17(5):1009. PMID: 29551741; PMCID: PMC6139072. 10.1016/j.cgh.2018.03.012 PMC6139072 29551741

[21] Behzadifar M, Gorji HA, Rezapour A, Bragazzi NL (2019). Comparison of prevention, screening and treatment of hepatitis C in Iran, Egypt and Georgia. J Virus Erad.

[22] Abdel-Aty M, Fouad M, Sallam MM, Elgohary EA, Ismael A, Nawara A, Hawary B, Tag-Adeen M, Khaled S (2017). Incidence of HCV induced-esophageal varices in Egypt: Valuable knowledge using data mining analysis. Med (Baltim).

[23] Amoako, Duah et al. Indications and findings of lower gastrointestinal endoscopy: a retrospective study in Eastern Regional Hospital, Koforidua, Ghana. (2020). PAMJ Clinical Medicine.;3(65).

[24] Alatise OI, Arigbabu AO, Agbakwuru EA, Lawal OO, Ndububa DA, Ojo OS (2012). Spectrum of colonoscopy findings in Ile-Ife Nigeria. Niger Postgrd Med J.

[25] Elbatea H, Enaba M, Elkassas G, El-Kalla F, Elfert AA (2011). Indications and outcome of colonoscopy in the middle of Nile delta of Egypt. Dig Dis Sci.

[26] Elkeleny MR, Abdelbaki TN, Sabry AA, Sharaan M (2021). Colonoscopic screening in early detection of colorectal cancer in high-risk groups: a prospective study. Egypt J Surg.

[27] Abu Aeshah W, Afifi S, Mohammed A, Sadek A (2021). Screening and Prevalence of Internal Hemorrhoids in patients undergoing flexible colonoscopy. Egypt J Hosp Med.

[28] Frontiers in medicine, 9, 867293. https://doi.org/10.3389/fmed.2022.867293.

[29] Le Kmieciak M, Gaudric M, Sogni P. Relevance of colonoscopy indications in an AP-HP Gastroenterology Department in 2001; application of Criteria established by a panel of European experts. (2003). Gastroentérologie Clinique et Biologique.;27: 213–8). 12658131

[30] Afify S, Tag-Adeen M, Abu-Elfatth A, Eid A, Nageh A, Alzamzamy A, El-Raey F, Basiony AN, Abdelghani M, Abdeltawab D, Ahmed RM, Nasr H, Alkady MN, Ibrahim W, Elshaarawy O, Amer H, Thoufeeq M, Alboraie M (2022). Quality indicators for colonoscopy in Egypt: a prospective multicenter study. Arab J Gastroenterology: Official Publication Pan-Arab Association Gastroenterol.

[31] Rees CJ, Gibson T, Rutter S, Baragwanath MD, Pullan P, Feeney R, Haslam M, British Society of Gastroenterology, the Joint Advisory Group on GI Endoscopy, the Association of Coloproctology of Great Britain and Ireland (2016). UK key performance indicators and quality assurance standards for colonoscopy. Gut.

