# Correction: Clinical and endoscopic characteristics of patients undergoing gastrointestinal endoscopic procedures in Egypt: a nationwide multicenter study

**DOI:** 10.1186/s12876-024-03281-0

**Published:** 2024-06-13

**Authors:** Mohamed Elbadry, Fathiya El-Raey, Mohamed Alboraie, Mohamed Abdel-Samiee, Doaa Abdeltawab, Mohammed Hussien Ahmed, Ahmed F Sherief, Ahmed Eliwa, Mina Tharwat, Amira Abdelmawgod, Ossama Ashraf Ahmed, Eman Abdelsameea, Aya Mahros, Abdelmajeed M. Moussa, Alshaimaa Eid, Khaled Raafat, Ahmed Yousef, Saad A. S. Rafea, Youssef Alazzaq, Mohamed Mare’y, Ahmed Abdelaziz, El Sayed Abouzid Ibrahim, Waleed A. Abd El Dayem, Ahmed A Abdelmoati, Ahmed Tawheed, Mohammad Amer, Samy Zaky, Mohamed El-Kassas

**Affiliations:** 1 Endemic Medicine Department, Faculty of Medicine, Helwan University, Ain Helwan, Cairo, 11795 Egypt; 2 Hepatogastroenterology and Infectious Diseases Department, Al-Azhar University, Damietta, Egypt; 3 Department of Internal Medicine, Al-Azhar University, Cairo, Egypt; 4 Hepatology and Gastroenterology Department, National Liver Institute, Menoufia University, Shebin El-Kom, Egypt; 5 Tropical Medicine and Gastroenterology Department, Assiut University, Assiut, Egypt; 6 Hepatology, Gastroenterology, and Infectious Diseases Department, Kafrelsheikh University, Kafrelsheikh, Egypt; 7 Tropical Medicine Department, Ain Shams University, Cairo, Egypt; 8 Tropical Medicine and Gastroenterology Department, Aswan University, Aswan, Egypt; 9 Internal Medicine Department, Ain Shams University, Cairo, Egypt; 10 Hepatogastroenterolgy and Infectious Diseases Department, AL-Azhar University, Cairo, Egypt; 11 Public Health and Community Medicine Department, Damietta Faculty of Medicine, Al-Azhar University, Damietta, Egypt; 12 Faculty of Medicine, Al-Azhar University, Damietta, Egypt; 13 Medicine Department, Al-Azhar University, Damietta, Egypt; 14 Tropical Medicine Department, Zagazig University, Zagazig, Egypt; 15 Hepatology & Gastroenterology, Shebin Teaching Hospital, Shebin El Kom, Egypt

**Correction: BMC Gastroenterol 24, 186 (2024)**

10.1186/s12876-024-03262-3

Following publication of the original article it was reported that due to a typesetting error there was an error in the city listed in Affiliation 3. The city was incorrectly given as Damietta but should be Cairo. Furthermore, the department was incorrectly given as ‘Internal Medicine Department’ instead of ‘Department of Internal Medicine’.

The correct affiliations are given in this Correction article and the original article has been updated.

